# “Us and Them”: a social network analysis of physicians’ professional networks and their attitudes towards EBM

**DOI:** 10.1186/1472-6963-13-429

**Published:** 2013-10-22

**Authors:** Daniele Mascia, Americo Cicchetti, Gianfranco Damiani

**Affiliations:** 1 Catholic University of the Sacred Heart, Department of Public Health, Largo Francesco Vito 1, Rome 00168, Italy; 2 Catholic University of the Sacred Heart, SEGESTA Department of Management, Largo Francesco Vito 1, Rome 00168, Italy

**Keywords:** Evidence-Based Medicine, Hospital organizations, Social networks, Core-periphery analysis, Structural importance

## Abstract

**Background:**

Extant research suggests that there is a strong social component to Evidence-Based Medicine (EBM) adoption since professional networks amongst physicians are strongly associated with their attitudes towards EBM. Despite this evidence, it is still unknown whether individual attitudes to use scientific evidence in clinical decision-making influence the position that physicians hold in their professional network. This paper explores how physicians’ attitudes towards EBM is related to the network position they occupy within healthcare organizations.

**Methods:**

Data pertain to a sample of Italian physicians, whose professional network relationships, demographics and work-profile characteristics were collected. A social network analysis was performed to capture the structural importance of physicians in the collaboration network by the means of a core-periphery analysis and the computation of network centrality indicators. Then, regression analysis was used to test the association between the network position of individual clinicians and their attitudes towards EBM.

**Results:**

Findings documented that the overall network structure is made up of a dense cohesive core of physicians and of less connected clinicians who occupy the periphery. A negative association between the physicians’ attitudes towards EBM and the coreness they exhibited in the professional network was also found. Network centrality indicators confirmed these results documenting a negative association between physicians’ propensity to use EBM and their structural importance in the professional network.

**Conclusions:**

Attitudes that physicians show towards EBM are related to the part (core or periphery) of the professional networks to which they belong as well as to their structural importance. By identifying virtuous attitudes and behaviors of professionals within their organizations, policymakers and executives may avoid marginalization and stimulate integration and continuity of care, both within and across the boundaries of healthcare providers.

## Background

Prior literature has widely documented that there is a significant association between the propensity of physicians to use Evidence-Based Medicine (EBM) in their practice and the structural characteristics of their professional networks [1,2]. In particular, this stream of research has shown that the network characteristics of professional relationships among clinicians are important predictors in explaining their different orientation towards EBM [2-4].

Although EBM has been widely considered as an individual attitude, its actual impact within organizations strongly relies on its pervasiveness and widespread diffusion at the organizational level [5]. If physicians do practice EBM individually, the risk is that barriers to the effective implementation of innovative clinical solutions are not translated “from the bench to the bed” of the patient [6,7]. These difficulties are often due to social constraints and barriers which some elitists may establish against other non-elitarian members within organizations [8], as well as the resistance of other clinicians who have a different behavioral orientation [9,10].

Despite its general importance, this topic has been seldom analyzed on empirical grounds in healthcare organizations. The aim of the present paper is to fill this gap by exploring and testing whether physicians’ self-reported frequency of EBM adoption is related to the network position they hold in the overall web of collaborative relationships established within healthcare organizations, where they routinely visit and treat their patients.

Data regarding a community of hospital physicians staffed in one of the biggest Italian healthcare organizations were collected and used in the present study. Social network analysis was firstly performed to identify structurally important physicians in the network. Specifically, we derived a core-periphery structure of the overall inter-physician network, distinguishing the dense cohesive core of the professional network from the sparse, unconnected periphery. Then, a new class of network centrality indicators, overall called Hubs and Authorities centrality, were employed to capture the structural prominence that physicians exhibited in the network. Finally, we explored whether their self-reported frequency of EBM adoption predicted the degree of coreness and structural importance that individual doctors assume within the observed network.

Social networks research has provided ample evidence that individuals’ attitudes and other personal characteristics influence the shape of their social networks as well as the position they assume in the overall web of relationships [11-13]. On the basis of previous work developed in this field [14], we assume that the propensity towards EBM is a relatively stable physician individual characteristic, which in turn influences his/her network position within organizations. We hypothesize that there is an association between the physicians’ propensity to use EBM and their degree of coreness within organizations, taking other relevant individual and organizational characteristics into consideration.

## Methods

### Research setting and data collection

The present observational study was conducted using a questionnaire survey of 329 physicians employed in six hospitals belonging to one of the largest Italian local health authorities (LHAs).

In Italy, LHAs aim to promote and protect the health of all resident citizens of a specific territory. The Italian National Health Service (INHS) is currently comprised of 145 LHAs. Based on considerations of efficiency and cost-effectiveness, each LHA may provide direct care through its own facilities or may commission the services to providers accredited by the system, such as independent public and private bodies.

The surveyed LHA serves approximately 800,000 individuals residing in 50 municipalities. The LHA employs around 8,400 people, including technical staff, nurses and physicians, and more than 80,000 hospitalizations occur annually.

Hospital activities are carried out according to a matrix organizational model. Although hospital activities are carried out in six hospital facilities, these hospital services are provided by three clinical directorates, which may be considered as the health sector equivalent of strategic business units [15]. Clinical directorates are managerially inspired and defined groupings of clinical specialties and support services created specifically for the purposes of resource management, control and accountability. They are intermediate organizational establishments through which defined parts of larger hospitals’ health services are managed. The directorates were introduced in the Italian healthcare organizations in the 1990s (laws 502/1992 and 229/1999), with the aim of reorienting activities toward healthcare processes [15].

Data were collected using a questionnaire, which was administered from February to November 2007. Participation was voluntary, and respondents were assured that their responses would be confidential and used for research purposes only. Because our study contains no experimental research, and given that any information concerning patients was collected, in accordance with Italian law ethics approval was not necessary. However, all physicians provided informed consent for the survey.

The questionnaire consisted of three sections, which contained a total of 17 questions. The first section collected attributive data on clinicians, such as: age, gender, hospital tenure, prior experience in the NHS and managerial role. The second section was designed to collect data on advice network relationships among clinicians. According to Burt’s approach [16], we used an egocentric social-network survey instrument to derive a list of people with whom the respondent had ties with. Each physician was asked to name colleagues within and outside their hospital organization with whom they interacted with through relationships based on the exchange of advice, and responses were combined in a summary network. Each respondent was asked to characterize tie strength with each nominated peer using a five-point scale. The third section of the questionnaire collected information about clinicians’ attitudes towards EBM. It included questions about respondents’ perceptions of the availability of information and the possibility of accessing scientific evidence through corporate information-technology support.

Responses to the questionnaire were requested within 3 months. Two quarterly recalls were sent to the physicians via email and a final recall asked for a response within 1 month. After ten months the questionnaire was activated and made available online, almost 90% of the total population (# 297 physicians) completed the questionnaire.

### Variables and measures

#### *Dependent variable*

Social network analysis was used to derive the position of individual physicians within the surveyed professional network. Using survey relational data, an adjacency (or square) matrix containing information on the interpersonal collaborative ties among clinicians was created [17]. Each row/column listed physicians surveyed and intersecting cells represented the frequency (intensity) of interaction between pairs of individuals. After data preparation, we used the continuous Core-Periphery algorithm developed by Borgatti and Everett [18] to compute the degree of coreness of each surveyed physician. As Borgatti and Everett clarify: “the core periphery model consists of two classes of nodes, namely a cohesive subgraph of the core, in which actors are connected to each other in some maximal sense and a class of actors that are more loosely connected to the cohesive subgraph but lack any maximal cohesion with the core.” [18:378] Core periphery algorithms jointly consider two kinds of structural properties of network nodes. First, the level of centrality that a given actor assumes within the network is considered. At the same time, the algorithms take into account the general level of interconnectedness it exhibits with other network nodes. A *Network Coreness* score was computed and then assigned to each sampled physician.

A Hubs and Authorities analysis was conducted to complement the core-periphery analysis described above. Hubs and Authorities analysis represents a natural generalization of the eigenvector centrality analysis, which can effectively identify the structural importance of individual actors in social networks [19,20]. A set of algorithms is defined to compute two distinct and heavily interwoven measures, called “hub” and “authority”, which reflect the prominence of each actor based on the structural characteristics of his/her network ties. An actor can be defined as highly hub central whether he/she points, i.e. he/she has many out-going ties, to many good authorities. High authority actors are those who receive, showing many in-coming ties, from many good hubs. Kleinberg clarifies [19] that “[t]he authority score of a vertex is proportional to the sum of the hub scores of the vertices on the in-coming ties and the hub score is proportional to the authority scores of the vertices on the out-going ties.” Overall, clinicians experiencing high *Network Authority* scores can be commonly regarded as important actors since they are both relevant and popular within the network.

The UCINET 6.392 software package was used in the present study to perform the analysis of the surveyed professional network [21].

#### *Independent variable*

As in previous research, physicians’ attitudes towards EBM (*EBM adoption*) were investigated by asking how often in the past year they had used scientific evidence published in peer-reviewed biomedical journals to aid their medical practice [2,14,22,23]. The survey questionnaire specifically asked individuals to answer “How often did you use scientific evidence published in peer-reviewed biomedical journals in your medical practice over the last year?”. Responses were rated on a 4-point Likert-scale structured as follows: “never,” “rarely,” “sometimes,” and “often/very often”.

#### *Control variables*

A number of other demographic and work-profile variables that might affect the position that physicians occupy within the organizational network were considered and included in the regression models. Some attributive characteristics of each physician were included, such as: *Age*, *Gender* and years of prior experience within the INHS (*Tenure INHS*) and within the LHA (*Tenure LHA*). A dummy variable that considered the managerial responsibility (*Managerial Role*) of each physician was assigned a value of 1 if the physician played a managerial role within the hospital system or 0 otherwise. Given that the geographical distance for other colleagues likely affects the possibility of interaction between them, and thus the position that an individual occupies within the network, a variable named *Geographical Proximity* was computed as the reciprocal of the average geographical distance (expressed in kilometers) of each sampled physician from their organizational colleagues. Finally, a set of dummy variables that considers physicians’ affiliation to the various LHA hospitals and directorates was entered into the model.

## Results

The overall sample is made up of 297 physicians. Table 1 shows the main characteristics of sampled individuals. They are, on average, 47 years old (SD 8.01) and are mostly men. The number of years they have accumulated in the INHS is, on average, 16.01 (SD 8.01). Years of experience that have been accumulated within the organization is, instead, 10.95 (SD 7.94). Only 51 physicians are clinical managers. As for EBM adoption, the majority (71.5%) reported adopting EBM frequently, followed by physicians declaring to adopt EBM very frequently (14.49%), occasionally (12.56%) and never (1.45%). Overall, almost 86% of sampled physicians reported to adopt EBM frequently or very frequently.

**Table 1:** Characteristics of sampled physicians (N = 297)

Network Coreness, mean ± SD (range)	0.053 ± 0.024 (0.011 – 0.125)
Network Authority, mean ± SD (range)	0.042 ± 0.049 (0.000 – 0.195)
EBM adoption, No. Physicians (%)	
Very frequently	43 (14.49)
Frequently	212 (71.50)
Occasionally	37 (12.56)
Rarely	4 (1.45)
Age, year, mean ± SD (range)	47.01 ± 8.01 (30 – 67)
Gender, No. M/F	158/139
Tenure INHS, year, mean ± SD (range)	16.01 ± 9.74 (1 – 41)
Tenure LHA, year, mean ± SD (range)	10.95 ± 7.94 (1 – 37)
Managerial role, No. managers/professionals	51/246
Geographical distance, kilometers, mean ± SD (range)	22.77 ± 29.61 (0 – 198)
Hospital affiliation, No. Physicians (%)	
Hospital 1	6 (2.02)
Hospital 2	20 (6.73)
Hospital 3	9 (3.03)
Hospital 4	112 (37.31)
Hospital 5	121 (40.74)
Hospital 6	29 (9.76)
Directorate affiliation, No. Physicians (%)	
Directorate 1	48 (16.16)
Directorate 2	75 (25.25)
Directorate 3	174 (58.59)

**Figure 1:**
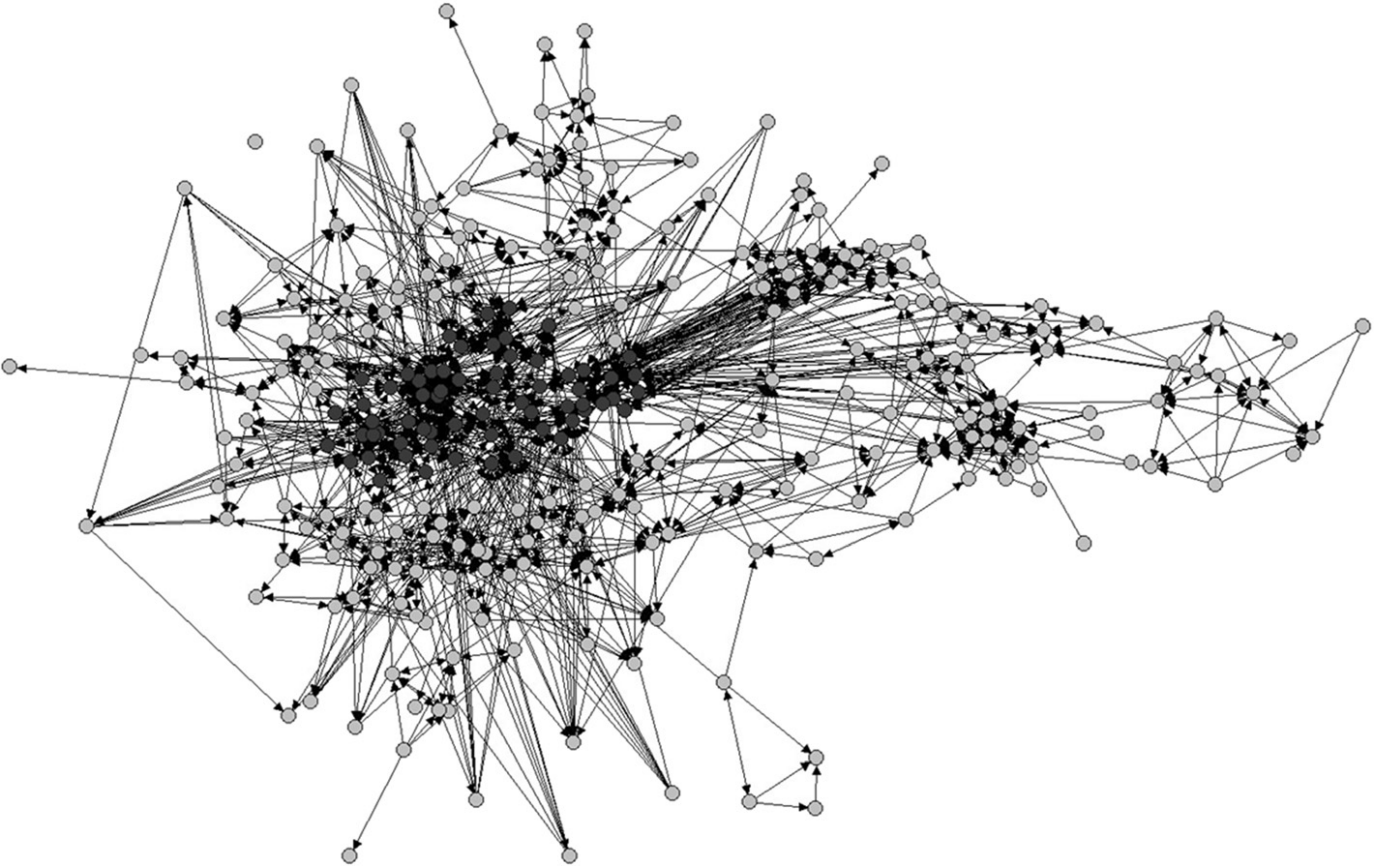
**Sociogram of the professional network among physicians.** Legend: Number of nodes = 297; Number of ties = 1,473; Network density (proportion of existing ties relative to the number of possible ties) = 5.7%. Dark gray circles represent physicians who are part of the network core and light gray circles are clinicians who belong to the network periphery.

Figure 1 illustrates the network of collaborative relationships among sampled physicians. The circle (node) represents physician and the link (edge) represents an existing collaborative tie among node pairs. Physicians' locations in Figure 1 were determined using a spring-embedding heuristic, multidimensional scaling algorithm, with proximity indicating the extent to which two clinicians were connected directly and indirectly through mutual colleagues [21].

Table 2 shows the pairwise correlations among variables. The inspection of coefficients reveals a strong and positive association between age, tenure in the INHS, and organizational tenure. Tenure in the INHS is, in turn, positively associated to the variable making distinction of whether the clinician has a managerial role or not. Network coreness and network authority variables showed to be moderately associated with both the propensity to adopt EBM and geographical proximity of physicians, albeit with a different sign.

**Table 2:** Pairwise correlations among variables (N = 297)

	**Variable**	** 1**	** 2**	** 3**	** 4**	** 5**	** 6**	** 7**	** 8**	** 9**	** 10**	** 11**	** 12**	** 13**	** 14**	** 15**
1	Network Coreness	-														
2	Network Authority	0.412	-													
3	EBM adoption	-0.566	-0.326	-												
4	Age	0.056	0.077	-0.058	-											
5	Gender	0.018	-0.034	0.016	-0.180	-										
6	Tenure INHS	0.068	0.081	-0.176	0.874	-0.150	-									
7	Tenure LHA	0.017	0.018	-0.184	0.691	-0.109	0.780	-								
8	Managerial role	0.144	0.147	0.080	0.453	-0.232	0.490	0.309	-							
9	Geogr. proximity	0.417	0.310	0.063	0.125	-0.081	0.139	0.063	0.126	-						
10	Hospital 2	-0.190	-0.123	0.053	-0.123	0.014	-0.140	-0.135	-0.070	-0.102	-					
11	Hospital 3	-0.201	-0.135	-0.090	-0.013	-0.119	-0.002	-0.062	-0.076	-0.118	-0.032	-				
12	Hospital 4	0.000	-0.311	0.066	-0.096	0.110	-0.125	-0.117	0.010	-0.034	-0.122	-0.132	-			
13	Hospital 5	-0.022	0.087	-0.068	0.132	-0.086	0.159	0.269	0.032	0.041	-0.165	-0.179	-0.677	-		
14	Hospital 6	0.179	0.346	0.033	0.031	0.045	0.020	-0.129	0.034	0.136	-0.061	-0.066	-0.250	-0.339	-	
15	Directorate 2	0.116	-0.246	-0.025	0.000	0.060	0.028	-0.008	-0.125	-0.150	0.253	0.037	0.182	-0.296	-0.109	-
16	Directorate 3	0.019	0.333	-0.088	-0.015	-0.058	-0.067	-0.090	0.069	0.176	-0.302	0.045	0.095	-0.081	0.202	-0.540

Tables 3 and 4 show the OLS (Ordinary Least Squares) regression results. Stata 10 was used to perform the regression analysis.

**Table 3:** OLS regression predicting the Network Coreness of physicians within the network

	**Model M1**		**Model M2**		**Model M3**	
Intercept	0.063 (0.007)	***	0.071 (0.015)	***	0.085 (0.017)	***
EBM adoption	-0.004 (0.002)	**			-0.006 (0.003)	**
Age			0.000 (0.000)		0.000 (0.000)	
Gender			0.002 (0.003)		0.003 (0.003)	
Tenure INHS			-0.000 (0.000)		-0.001 (0.000)	
Tenure LHA			0.000 (0.000)		0.000 (0.000)	
Managerial role			0.011 (0.005)	**	0.013 (0.005)	**
Geogr. proximity			0.001 (0.000)	***	0.001 (0.000)	***
Hospital 1			(omitted)		(omitted)	
Hospital 2			-0.056 (0.007)	***	-0.057 (0.007)	***
Hospital 3			-0.045 (0.009)	***	-0.047 (0.008)	***
Hospital 4			-0.029 (0.008)	***	-0.029 (0.008)	***
Hospital 5			-0.026 (0.008)	***	-0.027 (0.008)	***
Hospital 6			-0.017 (0.009)	*	-0.018 (0.009)	**
Directorate 1			(omitted)		(omitted)	
Directorate 2			0.020 (0.007)	***	0.019 (0.007)	**
Directorate 3			-0.001 (0.004)		-0.001 (0.005)	
*Regr. Diagnostics*						
N. of obs.	297		297		297	
R-squared	0.070		0.306		0.342	
Prob > F	0.020		0.000		0.000	

Note: *** p < 0.01; ** p < 0.05; * p < 0.10; Robust standard error in parentheses; Standardized coefficients.

**Table 4:** OLS regression predicting the Network Authority of physicians within the network

	**Model M1**		**Model M2**		**Model M3**	
Intercept	0.040 (0.007)	***	0.013 (0.031)		0.025 (0.031)	
EBM adoption	-0.009 (0.005)	**			-0.019 (0.008)	**
Age			-0.000 (0.001)		0.000 (0.001)	
Gender			0.006 (0.006)		0.007 (0.006)	
Tenure INHS			0.000 (0.001)		0.000 (0.001)	
Tenure LHA			0.001 (0.001)		0.001 (0.001)	
Managerial role			0.020 (0.011)	*	0.023 (0.011)	**
Geogr. proximity			0.001 (0.000)	***	0.001 (0.000)	***
Hospital 1		(omitted)	(omitted)			
Hospital 2			0.016 (0.012)		0.013 (0.012)	
Hospital 3			-0.012 (0.008)		-0.017 (0.010)	**
Hospital 4			-0.005 (0.007)		-0.008 (0.007)	
Hospital 5			0.052 (0.010)	***	0.048 (0.011)	***
Hospital 6			0.020 (0.013)		0.018 (0.013)	
Directorate 1		(omitted)	(omitted)			
Directorate 2			0.007 (0.006)		0.007 (0.006)	
Directorate 3			-0.047 (0.009)	***	-0.047 (0.009)	***
*Regr. Diagnostics*						
N. of obs.	297		297		297	
R-squared	0.048		0.385		0.418	
Prob > F	0.029		0.000		0.000	

Note: *** p < 0.01; ** p < 0.05; * p < 0.10; Robust standard error in parentheses; Standardized coefficients.

Table 3, in particular, presents three different models that we built to explore the clinician’s coreness within the professional network. Model M1 contains only the EBM adoption variable, and it should be considered as a null model against which the explanatory power of the subsequent models can be compared to. Model M2 includes only control variables. Model M3 is the full model incorporating all explanatory variables.

Model M1 in Table 3 shows that there is a significant association between the EBM variable and the network position that physicians hold in their professional network. In particular, it was found that a negative association exists between the physicians’ attitudes towards EBM and their degree of coreness (β = -0.004; *p* < 0.05). The regression results also documented that, among all of the structural and characteristic variables included, the coreness of individual physicians in their professional network was associated with Managerial Role, Geographical Proximity and the variables reflecting the clinician’s affiliation to hospital structures and directorates. In particular, Managerial role (β = 0.011; *p* < 0.05) and Geographical Proximity (β = 0.001; *p* < 0.01) were positively associated with the coreness of professionals within the organization. Physicians in Department #2 were more likely to exhibit a higher coreness than those in Department #1 (β = 0.020; *p* < 0.01), which is the baseline category of the model. Physicians in hospital facilities #2 (β = -0.056; *p* < 0.01), #3 (β = -0.045; *p* < 0.01), #4 (β = -0.029; *p* < 0.01), #5 (β = 0.026; *p* < 0.01) and #6 (β = -0.017; *p* < 0.10) exhibited a lower coreness score than those working in hospital #1.

Compared to the variables of Model M1, Model M2 includes the measure characterizing the physicians’ attitudes towards EBM in clinical practice. This variable showed a significant negative association (β = -0.006; *p* < 0.05) with the dependent variable, documenting that there is a negative association between the propensity to use EBM and the coreness that physicians exhibit in the overall professional network. All significant control variables in Model M1 continued to maintain significance in Model M2. Finally, it is important to note that the inclusion of the EBM adoption variable increased the overall fit of Model M3 over Model M2.

Table 4 presents all models exploring the association between the EBM adoption variable and the Network Authority variable. Our model building follows, similarly to what previously presented, a stepwise approach. According to this logic, Model M1 contains only the EBM adoption variable, Model M2 includes only control variables, and Model M3 is the full model that incorporates all explanatory variables.

Models M1 and M3 in Table 4 document that a negative and significant association exists between the EBM adoption variable and the structural importance that physicians hold in the collaboration network (β = -0.009; *p* < 0.05 in M1; β = -0.019, *p* < 0.05 in M3). The inspection of parameters corresponding to control variables overall confirms our previous results documenting a significant association between the network centrality of clinicians and a number of other contingencies, such as their spatial proximity from colleagues (β = -0.001; *p* < 0.01 in M2; β = -0.001, *p* < 0.01 in M3), the managerial position they eventually occupy in the organization (β = 0.020; *p* < 0.1 in M2; β = 0.023, *p* < 0.05 in M3), and their belonging to specific hospitals (hospital #5, β = 0.052; *p* < 0.01 in M2; β = 0.048, *p* < 0.01 in M3) and departmental arrangements (Department #3, β = -0.047; *p* < 0.01 in M2; β = -0.047, *p* < 0.01 in M3).

## Discussion

EBM represents one of the most important paradigms in modern medicine [24-26]. Clinicians and healthcare professionals in general are requested to increasingly adopt and integrate the latest available medical knowledge produced in their clinical practice [27].

In this study, we explored how the propensity towards EBM is associated with the position that professionals occupy in the overall network of collaborative ties they create within healthcare organizations. Our findings documented that there is a significant negative association between the physicians’ propensity to use EBM and the coreness they exhibit in their organization. Our analysis also indicated that the core is formed by physicians having a significantly lower propensity towards EBM than their peers located in the peripheral part of the network. Supplementary analyses were performed to capture closely the structural importance of physicians in the professional network through the use of network centrality indicators. Our findings again provided evidence for a negative association between EBM adoption and the network prominence of individual clinicians.

Although the homophily of physicians in terms of EBM adoption has been documented elsewhere [23], in this study we show that higher EBM adoption can cause the isolation of such groups of professionals. Within organizations, there is the potential risk that professionals having this kind of behavior can be viewed as elitists who may behave in contrast with the practices routinely adopted within hospitals. As prior studies have shown [28], innovators within healthcare organizations often face hard times in changing the way consolidated practices are used daily. In particular, those who are located in the center of the network are less exposed to novelty and innovative behavior since their higher interconnectedness with homophilous pairs likely increases the risk to be influenced from colleagues [3].

Our findings are also consistent with extant research demonstrating that the acquisition of new knowledge by physicians often occurs more likely through personal relations than through explicit guidelines and clinical protocols [2]. Gabbay and le May [29] have shown that physicians often use mindlines instead of guidelines, because of their tendency to discuss clinical matters with colleagues instead of relying on documentation such as articles, meta-analysis and Cochrane library. In addition, the superior propensity towards EBM of clinicians forming the periphery might reduce their risk of becoming over-embedded.

Our findings have a number of implications. First, hospital executives are encouraged to identify groups of professionals that exhibit potentially virtuous attitudes and behaviors within their organizations. Social network analysis tools and techniques appear useful in this vein. They are, in addition, encouraged to foster collaboration across groups characterized by different propensities to use EBM in daily practices. The adoption of new organizational arrangements, processes and informal occasions of meeting, would all be useful means to achieve this objective. For example, increasing collaboration might be achieved through the internal restructuring of hospital organizations. The adoption of specific types of clinical directorates or interdisciplinary and interprofessional groups is an example in this direction [15,30]. New internal processes have to do with both organizational and professional streams of activities. Organizational processes for example concern the definition of objectives such us budget, quality standards and appropriateness, which may be targeted by administrators in order to encourage collaboration across heterogeneous groups. Finally, executives have the possibility to support the inclusion of medical leaders within organizations so that their role might be leveraged to persuade other professionals to collaborate more with EBM users [8].

Policy implications are also strong. Health systems around the world are urged to ensure coordination and integration amongst providers by fostering the collaboration of healthcare professionals belonging to different organizations. Policymakers may want to encourage healthcare administrators to implement the above mentioned actions. In addition, in this context, interorganizational cooperation may be better achieved by identifying EBM users in organizations and then leveraging the higher tendency to cooperate by the virtue of their homophily [23]. These initiatives might be, for instance, headed to foster a better continuity of care across organizations through the formation of interorganizational groups or the definition of innovative clinical pathways.

Our findings should be interpreted in light of several limitations. First, the degree of EBM adoption was self-reported in the present study. Although this is not an objective approach to studying physicians’ orientation to EBM, our approach seems to be consistent with extant research on this topic [2,5]. The type of study design poses another limitation. Given that all data were gathered at the same time, we cannot ascertain whether the collaboration of physicians with colleagues is an antecedent or consequence of EBM adoption. Even though the cross-sectional design adopted in this research prohibits us to determine causality, it provides however that a causal link exists between EBM utilization and social collaborative relationships. We encourage future longitudinal studies to disentangle the effect of physicians’ attitudes towards EBM and their propensity to establish collaborative ties in healthcare organizations.

## Conclusions

Our study documents that the overall network structure is made up of a dense cohesive core of physicians and a periphery made up of less connected clinicians. The social structure of this model underlies a group tightly connected physicians who interact strongly in order to exchange relevant knowledge, and a large number of less cohesive clinicians who are more likely to be connected amongst themselves than to members of the core part of the network. This result might be interpreted as a marginalization of physicians who are more prone to use EBM in their clinical practice.

This social structure may result in a fragmented organization, in which different habits and characteristics of groups of physicians likely increase the risk of conflicts and barriers for integration within hospital boundaries [31,32].

Social network analysis tools and techniques should be increasingly adopted by policymakers and administrators in order to support integration and coordination of clinical activities in complex social systems such as healthcare organizations.

## Abbreviations

EBM: Evidence-based medicine; INHS: Italian National Health Service; LHA: Local Health Authority.

## Competing interests

The authors declare that they have no competing interests in the present study.

## Authors’ contributions

DM and AC conceived the study and undertook the data collected. DM and GD contributed to the study design and performed the statistical analysis. All authors contributed to the subsequent manuscript drafts and approved the final manuscript.

## Pre-publication history

The pre-publication history for this paper can be accessed here:

http://www.biomedcentral.com/1472-6963/13/429/prepub
